# Editorial: Mechanisms of action of natural antisense transcripts on the post-transcriptional regulation of sense protein coding gene expression during development and in cancer

**DOI:** 10.3389/fmolb.2023.1268124

**Published:** 2023-08-30

**Authors:** Tominori Kimura, Gian Gaetano Tartaglia

**Affiliations:** ^1^ Laboratory of Microbiology and Cell Biology, Department of Pharmacy, College of Pharmaceutical Sciences, Ritsumeikan University, Kusatsu, Shiga, Japan; ^2^ Italian Institute of Technology (IIT), Geneva, Italy

**Keywords:** ncRNA, lncRNA, NAT, post-transcriptional regulation, A-form RNA:RNA duplex, protein-coding gene, cancer, development

The evolutionary development of an organism is associated with increased complexity of the regulatory potential of non-coding RNAs (ncRNAs), which constitute the majority of the transcriptome (Chowdhary et al., 2021). The most studied ncRNAs are microRNAs (miRNAs), which promote gene silencing by inhibiting translation of target genes and/or by destabilizing mRNAs (Lee et al., 1993; Wightman et al., 1993). In contrast, long ncRNAs (lncRNAs) are the least studied but the most complex group of ncRNAs. They have emerged to be more diverse than short ncRNAs and to have complex gene regulatory functions in cells. For instance, lncRNAs can form complex 3D secondary structures with the capacity to bind to proteins and nucleic acids (DNA and RNA). This dual capacity equips lncRNAs to be ideal regulators of the protein-nucleic acid network (Jadaliha et al., 2018).

Natural antisense transcripts (NATS) are lncRNAs that are transcribed from the opposite strand of protein coding genes and are complementary to or overlap the protein-coding transcript (Nishizawa et al., 2015). NATs are widespread in eukaryotic genomes with approximately 25%–50% of human and mouse gene loci being transcribed from the opposite as well as the forward strand (Katayama et al., 2005; Zhang et al., 2006). Consistently, an overwhelming number of NATs has been identified [https://www.gencodegenes.org, GENCODE Release (version30), Release date 04.2019], indicating the possibility that antisense-mediated regulation affects a large number of genes (He et al., 2008).

As part of this Research Topic, the comprehensive review by Khorkova et al. summarizes NAT-dependent post-transcriptional regulation of gene expression in a wide variety of biological mechanisms:

(i) Multiple NATs have been shown to regulate mRNA degradation or translation through sponging endogenous miRNAs. Wang et al. explored the role of MEF2C antisense RNA1 (MEF2C-AS1) on sponging miR-592 in cervical cancer. They found that MEF2C-AS1 was downregulated in cervical cancer and may regulate miR-592 and its target, the R-spondin1 axis, to affect cervical cancer cell invasion and migration. Liao et al. investigated the role of the lncRNA, NEAT1, in high glucose-induced hypertrophy of mesangial cells. They found that high glucose leads to STAT3 activation, resulting in downregulation of miR-222-3p by NEAT1, which acts as an miR-sponge. In this manner, NEAT1 limited miR-222-3p binding with CDK1B mRNA. The release of mRNA leads to elevated CDK1B protein levels, resulting in mesangial cell hypertrophy, which can cause diabetic nephropathy.
(ii) NATs bind their mRNA partner, thereby modulating its half-life in a miRNA-independent manner. Short complementary regions within the sense RNA:NAT pair may promote intermolecular RNA:RNA interactions (Nishizawa et al., 2015). These interactions are transient and unstable because of the low melting temperature of the small RNA duplex. The formation of this A-form RNA:RNA duplex triggers conformational changes in the sense RNA, allowing either enhanced accessibility of a stabilizing RNA-binding protein or decreased affinity of an RNA decay factor to the mRNA, thereby modulating its stability (Nishizawa et al., 2015; Jadaliha et al., 2018). Regulation of transcript stability can also be mediated by NAT-sponging of protein factors that regulate mRNA degradation (Pu et al., 2021).
(iii) Mammalian-wide interspersed repeat (MIR)-NATs mediate translational repression. NATs with embedded retrotransposon-derived repeats, such as MIRs, termed MIR-NATs, form effector domains that regulate mRNA-ribosome pairing. MIR-NATs overlapping 5′-UTRs head-to-head compete with internal ribosome entry sites (IRESs) for the 40S ribosome subunit and repress translation (Simone et al., 2021).
(iv) SINEUPs modulate translation initiation. SINEUPs (inverted SINEB2 sequence-mediated upregulating molecules) are encoded antisense to the 5′ end of the target sense mRNA and can enhance its translation without upregulating mRNA levels. The binding domain of SINEUPs is formed by the antisense region overlapping the start codon of the target mRNA and confers specificity to the protein coding transcript. An effector domain at the 3′ end of a SINEUP comprises embedded transposable element sequences, such as inverted short interspersed nuclear element B2, which is capable of upregulating translation by binding activating protein complexes (Podbevsek et al., 2018).
(v) Regulation by NAT-encoded mini-proteins. Short ORFs present in some NATs can be translated into peptides that have downstream regulatory functions in processes such as homeostasis regulation, disease pathogenesis, tumor oncogenesis, and development. Pan et al. review the latest developments in the field of lncRNA-encoded micropeptides.
(vi) Regulation of protein stability. NATs protect target proteins from degradation by sponging/inhibiting the activity of proteins involved in the ubiquitin-proteasome degradation pathway (Wang et al., 2021; Zhang et al., 2022).
(vii) Subcellular localization of proteins: NATs can also modulate the subcellular distribution of proteins (Suzuki et al., 2021), although this aspect of their activity has not been extensively studied.

As part of this Research Topic, Sirvinskas et al. report that increased expression of the antisense lncRNA, *CHROMR*, was associated with the development of malignancy and poor prognosis in brain glioma patients. However, experimental clarification of the mechanism of *CHROMR* action is required to account for the prognostic efficacy of the mRNA *PRKRA*/lncRNA *CHROMR* ratio that the authors claimed for glioma patients.

The recent discovery of vast ncRNA-based regulatory networks has revealed that the tissue- and developmental-specific expression of ncRNAs is important for them to be viable indicators of cell physiological state. Dysregulation of these networks has been implicated in diseases, including cancer. lncRNAs, NATs in particular, play a significant role in the networks involved in disease-relevant mechanisms, working either post-transcriptionally, as described above, or transcriptionally and epigenetically. In this Research Topic, Khorkova et al. fully describe these NAT-mediated biological mechanisms and highlight the recent clinical and pre-clinical developments in nucleic acid-based therapeutics that aim to modulate dysregulated ncRNA-based networks. This approach has opened a new set of therapeutic targets that were previously inaccessible to traditional protein-targeted small molecule inhibitors and will enable numerous new therapeutic possibilities. We hope readers of this Research Topic share our excitement in reading these articles that describe the recent advances in Regulatory RNA research.
